# The Role of Rab Proteins in Parkinson’s Disease Synaptopathy

**DOI:** 10.3390/biomedicines10081941

**Published:** 2022-08-10

**Authors:** Arianna Bellucci, Francesca Longhena, Maria Grazia Spillantini

**Affiliations:** 1 Department of Molecular and Translational Medicine, University of Brescia, 25123 Brescia, Italy; 2 Department of Clinical Neurosciences, University of Cambridge, Clifford Albutt Building, Cambridge CB2 0AH, UK

**Keywords:** Rab proteins, Parkinson’s disease, synaptopathy, autophagy, alpha-synuclein, GBA1, LRRK2

## Abstract

In patients affected by Parkinson’s disease (PD), the most common neurodegenerative movement disorder, the brain is characterized by the loss of dopaminergic neurons in the nigrostriatal system, leading to dyshomeostasis of the basal ganglia network activity that is linked to motility dysfunction. PD mostly arises as an age-associated sporadic disease, but several genetic forms also exist. Compelling evidence supports that synaptic damage and dysfunction characterize the very early phases of either sporadic or genetic forms of PD and that this early PD synaptopathy drives retrograde terminal-to-cell body degeneration, culminating in neuronal loss. The Ras-associated binding protein (Rab) family of small GTPases, which is involved in the maintenance of neuronal vesicular trafficking, synaptic architecture and function in the central nervous system, has recently emerged among the major players in PD synaptopathy. In this manuscript, we provide an overview of the main findings supporting the involvement of Rabs in either sporadic or genetic PD pathophysiology, and we highlight how Rab alterations participate in the onset of early synaptic damage and dysfunction.

## 1. Introduction

Parkinson’s disease (PD) is the most common neurodegenerative disorder with motor symptoms. It mostly arises as an age-associated sporadic disease, but several genetic forms also exist [1,2]. The brain of affected patients is characterized by the prominent loss of nigrostriatal dopaminergic neurons [3] and the presence of Lewy bodies (LB) and Lewy neurites (LN). These are insoluble proteinaceous deposits that form in neuronal cell bodies and processes, respectively, and are mainly composed of alpha-synuclein (aSyn) [4,5], a protein involved in the regulation of synaptic functions [6,7,8]. Indeed, small synaptic alpha-synuclein aggregates have also been reported in synaptic terminals in patients with synucleinopathies [9,10]. The loss of nigrostriatal dopaminergic neurons is the main culprit in the onset of motor symptoms in PD patients. Interestingly, human brain imaging studies highlighted that in both sporadic and genetic cases of PD, the loss of nigrostriatal neurons proceeds retrogradely, first involving putaminal synaptic terminals and projections and later affecting nigral cell bodies [11,12,13]. It has been estimated that, given the massive arborization of striatal axonal projections, each nigral dopaminergic neuron gives rise to about 2 million synapses in the putamen [14]. Therefore, the retrograde degeneration pattern of this neuronal population can account for the fact that the large number of connections established by nigrostriatal neurons guarantees a very high threshold of resilience to terminal loss. Along this line, a large body of evidence supports that motor disturbances appear when 80% of putaminal dopaminergic terminals are lost [12]. It is thus clear that synaptopathy plays a key role in triggering nigrostriatal dopaminergic neuronal loss in PD.

Interestingly, the analysis of post-mortem brains of patients affected by PD highlighted that the amount of aSyn deposited within synaptic microaggregates is several orders of magnitude higher than the actual amount of the protein within LB and LN [10]. This is in agreement with the fact that aSyn levels are enriched at synaptic terminals even in physiological conditions [4,6,7,12]. Abnormal accumulation of aSyn at synaptic terminals may thus promote pathological aSyn aggregates, initiating synaptic damage. Consistently, age-related dysfunctions in the homeostatic control of aSyn levels at synapses, such as alterations in the ubiquitin–proteasome system (UPS) and the autophagy–lysosome pathway (ALP), may prompt aSyn accumulation at synaptic terminals and consequently its pathological aggregation [15,16,17]. Moreover, multiplications of the aSyn gene (SNCA) cause the onset of a rare form of early-onset PD with rapid progression [18]. These findings support that the progressive accumulation of aSyn microaggregates at synaptic terminals represents a main toxic event contributing to neurodegeneration and disease progression in PD, while LB and LN may be a later evolutionary state of the microaggregates, deriving from the progression of the pathological deposition of the protein and the consequent impairment of proper aSyn trafficking at synapses. The main contribution of synaptic aSyn microaggregates to neuronal damage is also in line with the trans-synaptic spreading hypothesis of disease-relevant aSyn strains, which are believed to propagate pathology and disease symptoms in PD and other synucleinopathies [19]. Indeed, even though the mechanisms involved in the trans-synaptic conveyance of aSyn strains remain to be elucidated, it is feasible that the released toxic polymorphs could propagate neuronal damage either by affecting the integrity of outer synaptic membranes or by promoting aSyn aggregation in recipient neurons.

This notwithstanding, a plethora of genetic forms of PD linked to gene loci that are completely unrelated to aSyn also exist [20]. In some cases, these forms do not present LB or LN, and the incurring disease is defined as parkinsonism in that, neuropathologically, PD is defined by the presence of LB. Some of the genes mutated in PD encode for proteins involved in synaptic functions, such as: Ras-associated binding protein 39b (Rab39b), synaptojanin 1 (SYNJ1), Leucine-rich repeat kinase 2 (LRRK2), synphilin-1 (SYPH1) and transmembrane protein 230 (TMEM230) [21,22,23,24,25,26,27,28,29,30,31,32,33]. On the other hand, several PD-associated genes, including those encoding for some of the aforementioned proteins (RAB39B, SYPH1, SYNJ1 and TMEM230, in addition to GBA1, Parkin, PINK-1, ATP13A2, FBXO7 and SYT11), have been linked to dysregulations of ALP [31,32,34,35,36,37,38,39,40,41,42,43,44,45,46,47,48,49,50,51,52].

This scenario suggests that intracellular trafficking problems characterize PD, and they can be linked to synaptic derangement or ALP dysfunction [53]. Functionally intact anterograde and retrograde intracellular trafficking is crucial for ensuring and sustaining synaptic activity, and this is predicted to be especially relevant in nigrostriatal dopaminergic neurons, which are long-projecting and bear extensive arborization. Interestingly, alterations in several members of the Rab family of small GTPases (Rabs), key regulators of intracellular trafficking [54,55], have been reported in PD brains or in experimental models of the disease [53]. For instance, Rab39b, whose gene mutation is associated with familial PD, controls anterograde trafficking in the cell body [29]. Several other Rabs, such as Rab1, Rab3, Rab5, Rab7Aa, Rab8a, Rab8b, Rab10, Rab11a, Rab13, Rab29, Rab32 and Rab35, can affect aSyn aggregation, propagation and clearance, co-localize with aSyn, LRRK2, PTEN-induced kinase 1 (PINK1), Parkin and TMEM230 or participate in their function [56,57]. Here, we present a perspective on how Rab alterations can contribute to the onset of synaptopathy in either sporadic or genetic PD.

## 2. Rabs and Synaptic Function

Rabs are essential regulators of membrane trafficking and orchestrate cell physiology by spatially and temporally controlling vesicle sorting, fission, tethering, docking and fusion through interactions with multiple effector proteins [58,59,60,61,62]. In particular, they play an essential role in defining the identity of subcellular membranes, thus governing membrane trafficking [61,63]. The key role of Rabs in cell physiology is also supported by the fact that more than 60 Rabs have been described in eukaryotes.

Rab proteins are activated upon GTP binding, while they are inactive when they associate with GDP. GTPase-activating proteins (GAP) and guanine nucleotide exchange factor (GEF) control the exchange of GDP with GTP and GTP hydrolysis, thus regulating Rab activation and inactivation. Moreover, small GTPases carrying a C-terminal farnesyl or geranylgeranyl group, guanine dissociation inhibitors (GDIs), can form soluble complexes with small GTPases to combine the cytosol/membrane and GDP/GTP alternation [64]. Rab-GDI dissociation is then mediated by the GDI displacement factor (GDF) [65]. Importantly, GTP binding activates the Rab protein by leading to a major conformational modification in the so-called switch I and switch II regions, which mediate the binding to effector proteins [66].

Numerous studies have described the involvement of Rabs in the regulation of neurotransmitter release, ranging from the generation of synaptic vesicles (SVs), the control of their size and trafficking toward the synaptic cleft, and the mobilization, docking and recycling of SV pools at terminals to the retrograde transport of SVs toward the endosomal/lysosomal system. These processes can be regulated by a specific subset of Rabs, which include Rab3, Rab5, Rab11, Rab22, Rab26, Rab27b, Rab33 and Rab35 [67,68]. In particular, the control of SV release appears to be specifically regulated by Rab3a and Rab27b [69,70], both of which localize to SVs, bear elevated structural and functional redundancy and share common sets of regulators [71,72,73,74]. In agreement, Rab3-GTP can bind to SV membranes and dissociates from them upon GTP hydrolysis during neurotransmitter release [69]. Furthermore, Rab27b and Rab3a synergistically promote excitatory transmission in mammals [75].

Interestingly, rabphilin and Rab3-interacting molecule (RIM) represent the main Rab effectors in the control of SV exocytosis [58,71], though only the latter appears to play a relevant role in the regulation of neurotransmitter release [76,77], which is not significantly perturbed by rabphilin deficiency [78]. Indeed, RIM localizes to the active zone, where it forms a multimolecular complex with Rab3 and Munc13 [79,80]. While the RIM-Munc13 complex controls SV docking and priming and Ca^2+^-evoked release, RIM can also regulate both SV localization and release probability by a Munc13-independent-mechanism [81].

**Figure 1:**
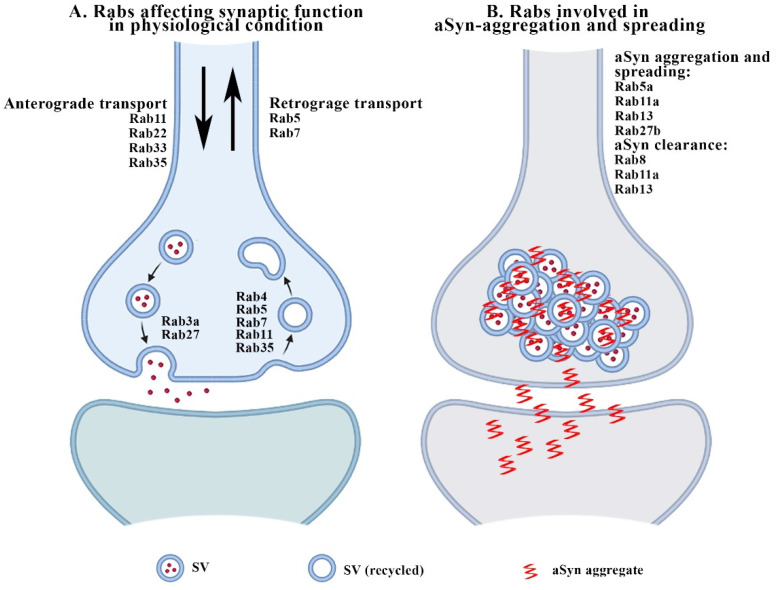
The cartoon summarizes the Rabs involved in the modulation of synaptic function in physiological conditions (**A**) and in aSyn aggregation and spreading (**B**).

Rab4, Rab5, Rab7, Rab11 and Rab35 control SV recycling [55]. In particular, Rab5 plays a primary role, as it controls the size and retrieval of SVs [82,83] and associates with the synaptic endosomal compartment [84,85]. While Rab11, Rab22, Rab33 and Rab35 are involved in the anterograde axonal transport of receptors, Rab5 and Rab7 play a major role in the control of retrograde axonal trafficking [68]. Rabphilin can interact with the Glutamate N2A receptor subunit (GluN2A)/postsynaptic density protein 95 (PSD-95) complex to retain N-methyl-D-aspartate (NMDA) receptors at synaptic sites, thus impinging on long-term potentiation (LTP)-dependent spine remodeling [86,87]. Rab 11 is involved in dendritic spine formation [88]. Finally, Rab26 and Rab33 have been proposed to be pivotal in SV degradation and turnover, as they are involved in the formation of the autophagosome [89,90] or in anterograde vesicular transport [91]. For a detailed summary of the role of Rabs in the modulation of synaptic function, please see Figure 1A.

## 3. Major Rab Alterations in PD and Other Forms of Parkinsonism: A Focus on aSyn-, GBA1- and LRRK2-Associated Synaptopathy

### 3.1. Rabs and aSyn-Related Synaptopathy

In 2014, Wilson and co-authors described that the c.503C > A missense mutation in RAB39B causes X-linked intellectual disability and early-onset PD with aSyn pathology as well as extensive dopaminergic neuronal loss in the substantia nigra [92]. By using both in silico modeling and site-directed mutagenesis, the authors found that this mutation destabilizes Rab39b, supporting its loss of function. Interestingly, they also described that the short-hairpin (sh)-RNA-mediated knockdown of RAB39B reduced the density of aSyn levels and immunoreactive puncta in the dendritic processes of cultured neurons. Moreover, the study reported the presence of axonal spheroids, structures reminiscent of the Wallerian-like degeneration observed in neurodegenerative diseases with impaired axonal transport [93], in the white matter tracts of the basal ganglia of the post-mortem brain of a patient bearing the c.503C > A RAB39B mutation [92]. This is in agreement with the involvement of Rab39b in endocytic retrograde and/or early-stage anterograde secretory transport [32].

Subsequently, other RAB39B mutations were reported to be associated with the onset of parkinsonism [94,95,96,97,98,99]. These can result in the total loss of Rab39B expression or in reduced Rab39b protein stability or altered function. Moreover, marked accumulation of Rab39b in Amyloid β (Aβ) plaques and a subset of LB in parallel with a marked reduction in Rab39b in the white matter regions were reported in the brains of patients affected by dementia with LB (DLB) [31].

Abnormal interaction of aSyn with rabphilin and the loss of rabphilin/Rab3a coupling have also been detected in PD brains [100]. Since aSyn, Rab3a and rabphilin play a relevant role in the regulation of SV release, it can be inferred that such alterations may contribute to impairing neurotransmitter exocytosis in LB diseases. Furthermore, the serum levels of Rab35 positively correlate with those of aSyn in PD patients, and the combined assessment of Rab35 and aSyn is a better predictive biomarker for discriminating PD patients from those affected by atypical parkinsonism or from healthy subjects [101], emphasizing the critical interplay between Rabs and aSyn. Rabs, particularly Rab5 with its effector Rabaptin-5, have been found in glial cytoplasmic inclusions (GCI), characteristic aggregates in the brains of multiple system atrophy (MSA) patients, also composed of aSyn [102,103].

The interaction between Rabs and aSyn is also supported by results from a plethora of studies on experimental models, where aSyn accumulation has been found to negatively impact Rab-mediated ER–Golgi homeostasis and SV fusion by affecting Rab1 and Rab3, respectively [104,105,106]. Moreover, overexpression of human A30P mutant aSyn in mice results in abnormal aSyn–Rab interaction [107].

On the other hand, Rabs were found to control the aggregation, toxicity and secretion of aSyn. Indeed, a recent microscopy-based large-scale RNA interference study showed that Rab8b, Rab11a, Rab13 and Rab39b modulate aSyn aggregation, toxic potential and levels [108]. In particular, Rab8b, Rab11a and Rab13 were found to promote the clearance of aSyn inclusions and rescue aSyn-induced toxicity. Rab11a and Rab13 expression also improved aSyn secretion and endocytic recycling in cells accumulating aSyn inclusions [108], while Rab3a recycling machinery and synaptic activity appear to control wild-type aSyn membrane association [109], and Rab5a-dependent aSyn endocytosis can induce neuronal death [110]. Finally, Rab27b has been reported to be relevant for aSyn aggregation, toxicity and spreading [111]. The Rabs possibly involved in aSyn aggregation and spreading at synaptic sites are listed in Figure 1B. A detailed summary of the alterations of Rabs related to aSyn synaptopathy is reported in Table 1.

### 3.2. Critical Involvement of Rabs in LRRK2-Associated Parkinsonism

Numerous mutations in the LRRK2 gene have been associated with late-onset autosomal-dominant PD [112]. Among them, the G2019S variant residing in the kinase domain is among the most common, together with R1441G, located in the ROC domain, and G2385R in the WD40 domain [113,114,115,116]. Rab1, Rab3, Rab5, Rab8, Rab10, Rab12, Rab29, Rab35 and Rab43 are among the most important LRRK2 substrates and mediate multiple LRRK2 functions [117]. Moreover, Rab29 recruits LRRK2 to the trans-Golgi to activate its kinase activity [118,119]. This in turn leads to Rab8, Rab10 and Rab12 phosphorylation [120,121]. However, since the basal phosphorylation of Rab10 and Rab12 by LRRK2 is not affected by the knockout or overexpression of Rab29 [122], it is unlikely that the encoded protein is among the mediators of basal or pathogenic LRRK2 phosphorylation activity.

Rab10 phosphorylation is differentially affected by the R1441C- and G2019S-LRRK2 mutations, with the former inducing an increase and the latter causing a decrease in the process [123]. The overexpression of pathological LRRK2 in LRRK2-mutant cells and fibroblasts from G2019S-LRRK2 PD patients decreases Rab7 activity and results in delayed endosomal trafficking and impaired epidermal growth factor receptor (EGFR) degradation, which can be reversed upon LRRK2 inhibition and overexpression of Rab7 [124]. However, Rab7 is not an LRRK2 phosphorylation substrate [117,125], and G2019S-LRRK2 patients do not exhibit alterations in Rab7 levels [124].

Moreover, concerning the Rabs involved in endolysosomal trafficking, it has been described that the loss of Rab8 mimics deficits in endolysosomal function and impairs EGFR degradation, similarly to Rab7 activity reduction. The loss of Rab8 also reduces Rab7 activity, suggesting a relationship between the two and indicating that Rab7 activity is located downstream from Rab8. Notably, in contrast to what has been observed for Rab7, the expression of active Rab8 or the up-regulation of the Rab11-rabin8 cascade rescues deficits in endolysosomal membrane trafficking mediated by G2019S-LRRK2 [126]. Rab35 is involved in endocytic recycling and is another substrate of LRRK2 [127]. It has been described that increased LRRK2 kinase activity enhances aSyn propagation through LRRK2-mediated Rab35 phosphorylation [128]. Consistently, the functional inhibition of LRRK2 kinase activity reduced both Rab35 and aSyn levels [128]. Whether the reduction in Rab35 levels results from an increase in its degradation or a reduction in its synthesis due to LRRK2 inhibition is not yet clear. Rab32 can directly interact with LRRK2, affecting retromer trafficking in the trans-Golgi [129,130]. LRRK2, Rab5 and Rab11 have been found to be involved in SV recycling in the *Drosophila* model [131].

Given the essential role exerted by LRRK2 in the regulation of synaptic function [21,132] and its interplay with Rabs, it is easily foreseeable that these proteins may together contribute to pathogenic LRRK2-associated synaptopathy. Indeed, LRRK2 kinase activity regulates SV trafficking, neurotransmitter release and synaptic morphology [26,133], and LRRK2 kinase inhibition also promotes anterograde axonal transport and presynaptic targeting of aSyn [134]. G2019S- or R1441C-LRRK2 mutants assist the activity of glutamatergic synapses thus inducing excitotoxic dendritic degeneration [135]. The G2019S-LRRK2 mutation induces dopaminergic nigral neuron dysfunction, affects corticostriatal long-term depression and also promotes tau spreading in the mouse brain [136,137]. Furthermore, though different LRRK2 kinase inhibitors do not perturb basal dopamine release or firing in nigrostriatal neurons in wild-type mice, GNE-7915, an LRRK2 inhibitor, enhanced dopamine release and SV mobilization and recycling in BAC LRRK2 hG2019S and hR1441G transgenic mice [138]. It can thus be speculated that since Rab5 and Rab11 are LRRK2 kinase substrates regulating SV recycling [131], they may be directly implicated in this effect and thus in pathogenic *LRRK2*-associated synaptopathy. It has been demonstrated that PD-associated LRRK2 mutants influence the degree of phosphorylation of different Rab proteins [139]. Among them, levels of Rab10 phosphorylation at threonine 73 (pT73) were found to be increased in peripheral blood mononuclear cells derived from heterozygous LRRK2 G2019S variant carriers [140] and in neutrophils and monocytes from patients carrying different LRRK2 mutations [141]. Moreover, Rab10 pT73 levels in urine exhibited a positive association with PD disease progression [142], suggesting that this could represent a valid target engagement biomarker for LRRK2 inhibitors in the clinic [143]. A decrease in Rab29 phosphorylation was also reported in urinary exosomes from patients with idiopathic and aSyn mutation-related PD [140]. Nevertheless, the indirect involvement of all LRRK2 kinase Rab substrates may also be predicted. Table 2 provides a summary of the involvement of Rabs in LRRK2 trafficking as well as in LRRK2-associated parkinsonism.

### 3.3. Involvement of Rabs in Other Genetic Forms of Parkinsonism

It has been described that several of the protein products of genetic parkinsonism-linked genes interact with Rabs. SYNJ1 regulates the endosomal trafficking of synaptic proteins by affecting Rab7 [144]. TMEM230, which plays a role in vesicle formation and trafficking, co-localizes with SVs and is also found in vesicular structures located in the perinuclear region, and it interacts with Rab5a, Rab7 and Rab11a [145]. TMEM230 is also required for Rab8a-mediated secretory vesicle and retromer trafficking [34].

Heterozygous mutations in the GBA1 gene, leading to glucocerebrosidase (GCase) dysfunction, are the most common genetic risk factor for PD [146,147]. Of note, reduced GCase activity can decrease lysosomal-mediated aSyn degradation, while aSyn inhibits GCase lysosomal activity, supporting that GCase deficiency and aSyn may set up a positive feedback loop propagating PD [148,149]. In agreement, reduced GCase activity can also occur in the brains of PD patients without GBA1 mutations and is associated with increased levels of phosphorylated aSyn, which is considered a marker of advanced LB pathology [149,150]. A reduction in GCase activity has also been shown to promote aSyn accumulation in different in vivo and in vitro models of PD [151,152]. For example, midbrain organoids derived from genetically engineered induced pluripotent stem cells with GBA1 deletion and SNCA overexpression develop LB-like inclusions [153]. However, it is also possible that a reduction in GCase activity may increase neuronal susceptibility to pre-existing aSyn aggregation [154]. Indeed, most of the subjects bearing either heterozygous or homozygous GBA1 mutations do not develop PD or aSyn aggregate deposition, supporting that other factors participate in linking reduced GCase activity to aSyn pathology [155,156]. For instance, GCase reduction can also impact the lipid compositions of late endosomal membranes and may thus consequently impair aSyn degradation through altered endosomal microautophagy or trigger aSyn oligomerization [157,158]. Indeed, the degradation of aSyn is reduced in lysosomes with reduced GCase enzymatic activity [159]. Furthermore, glycosphingolipid-induced aSyn accumulation can trigger cellular degeneration in human iPSC-derived midbrain neurons from patients with or without GBA1 mutations. On the contrary, glycosphingolipid-reducing agents improve synaptic localization of aSyn, reducing toxic aSyn assemblies in neuronal cultures [160]. It is also worth considering that different GBA1 mutations cause autophagy dysfunction [161,162], and Rabs are involved in various stages of autophagy (please see below for further details) [89,163]. In addition, Rab8 has also been suggested to promote the storage of lipids and lipid droplets [164]. It is thus plausible that Rab alterations are associated with GCase deficiency-related aSyn pathology and synaptopathy by modulating autophagy and/or lipid composition, two mechanisms implicated in GBA1 mutation-related PD synaptopathy [165]. In agreement, it has been reported that neuroinflammation and aSyn accumulation derived from GCase deficiency in mice are accompanied by synaptic dysfunction [166], which is also believed to be the starting point for disease progression in lysosomal storage disorders [167].

## 4. Interplay between Rabs and Autophagic Defects in PD Synaptopathy

Autophagy is a process leading to the self-degradation of unwanted or toxic macromolecules and organelles that are sequestered and delivered to the lysosome to generate raw materials (proteins, lipids, carbohydrates and nucleic acids) to be used in metabolic processes. Macroautophagy, chaperone-mediated autophagy (CMA) and microautophagy are the three main types of autophagy. CMA is a selective autophagic pathway that degrades cytosolic proteins with a particular pentapeptide motif upon their recognition by the chaperone Hsc70 and their subsequent entrance into lysosomes through the transmembrane protein lysosomal-associated membrane protein 2A (Lamp2A). Macroautophagy requires the formation of autophagosomes, double-membrane structures engulfing cytosolic components and degrading them through the generation of autophagolysosomes, which result from the fusion of autophagosomes and lysosomes. Finally, microautophagy degrades cytosolic components simply by invaginations in the lysosomal membrane. In post-mitotic neurons, autophagy is important for maintaining normal cellular homeostasis, particularly the critical turnover of misfolded proteins and damaged organelles [15,16].

**Figure 2:**
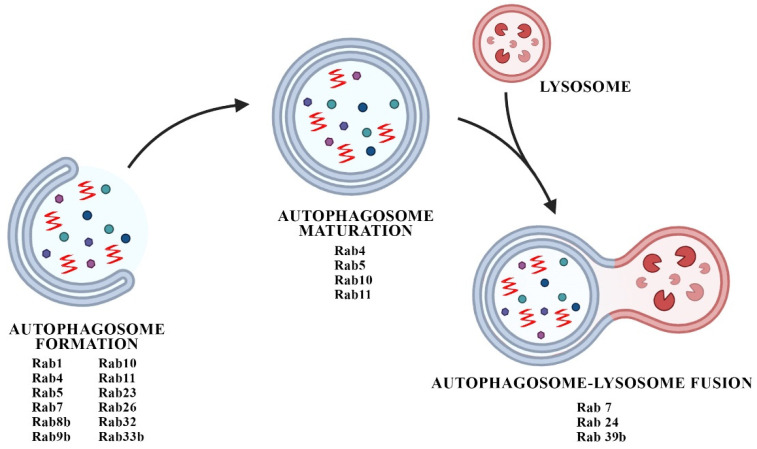
The cartoon summarizes the Rabs participating in the different phases of macroautophagy, from autophagosome formation to its fusion with the lysosome.

Rabs control different steps of autophagic processes but are mainly involved in the control of macroautophagy (Figure 2). Rab1, Rab5, Rab7, Rab9a, Rab11, Rab23, Rab32 and Rab33b participate in autophagosome formation, Rab9 is involved in non-canonical autophagy, and Rab7, Rab8b and Rab24 control autophagosome maturation [163]. Of note, the presence of large Rab7a-positive endosomes and an increase in Rab7a protein levels were detected in the post-mortem brains of patients affected by DLB [168]. Rab7 was also found to decrease aSyn pathology, probably by regulating autophagosome transport and fusion with lysosomes [169].

PARK2/Parkin mutations also affect the activity of Rabs by activating the effector proteins TBC1D15 and TBC1D17, which are required for routing damaged mitochondria to autophagosomes through Rab7 [170].

PINK1 was found to phosphorylate Ser111 of Rab8a, Rab8b and Rab13 in a Parkin-independent manner, thus impairing the interaction with GEF and consequently hampering Rab activation [171]. Parkin and PINK1 have proven involvement in the induction of mitophagy, the selective autophagic-based degradation of mitochondria [172,173], but recent evidence has also highlighted that PINK1, SYPH1 and seven in absentia homolog 1 (SIAH-1) complex constitutes a novel Parkin-independent mitophagy pathway [37], in which the possible involvement of Rabs can be postulated.

ATP13A2 encodes for a late endosomal/lysosomal ATPase, which modulates autophagy by regulating another PD-associated gene, synaptotagmin 11 (SYT11) [49]. The latter mediates a vesicle trafficking pathway that is essential for development and synaptic plasticity [174] and is also a mediator of Parkin-associated neurotoxicity [175]. Therefore, its interplay with Rabs and autophagy can be expected. GBA1 and LRRK2 mutations can affect Rabs and autophagy, as explained above, and RAB39B mutations can also impact autophagy [30,31].

Although the link between some PD pathological proteins and genes, Rabs, autophagy and synaptopathy is not fully clarified, autophagy is implicated in the control of synaptic function, and its alterations are expected to significantly impact synaptic homeostasis [176,177]. Synaptic proteins and SVs, postsynaptic receptors and synaptic mitochondria are known to be degraded by autophagy, thereby contributing to the remodeling of synapses [177]. Autophagy also regulates synaptic development [178], and autophagy modulation appears to be required for neurotransmission, different forms of synaptic plasticity and memory formation [176]. Not by chance, autophagosome formation is prominent at synaptic terminals, and neuronal autophagy is regulated in a compartment-specific fashion [176,179]. In turn, synaptic activity has been found to control dendritic autophagic vacuole motility and function [180]. There is an interdependency between autophagy and SV trafficking in the regulation of dopamine release, and this could involve Rab3 and Rab27 [181]. Finally, Rab26 links SVs to autophagic pathways [90].

Therefore, autophagy and synaptic dysfunction may promote a self-propagating disease pathway that compromises neuronal resilience, where Rabs can serve as critical mediators. Table 3 summarizes the involvement of Rabs in autophagy or autophagy-related synaptic alterations.

## 5. Conclusions

Altogether, these findings support that Rabs play a crucial role as direct or indirect (through autophagic involvement) mediators of synaptopathy in PD. Even though the involvement of Rabs in the induction of synaptic alterations concerning aSyn and LRRK2 is quite delineated, more studies are needed to decipher the interplay between many other PD genes, Rab alterations and synaptic pathologies. In particular, we envisage that more detailed investigations on experimental models of GBA1-, SYNJ1-, SYPH1-, TMEM230-, Parkin-, PINK1-, ATP13A2-, FBXO7- and SYT11-associated parkinsonism could provide new insight into the pathophysiological mechanisms leading to PD that may involve Rab dysfunction. Developing novel therapeutic strategies targeting Rabs could help to restore autophagic dysfunction and synaptopathy, thus counteracting the key mechanisms of neurodegeneration in PD.

## Figures and Tables

**Table 1:** Alterations of Rabs in aSyn-related synaptopathy.

**Rab Alterations and aSyn-Related Synaptopathy**		
**Rab**	**Alteration Type**	**References**
	**Mutations Identified in Rabs**	
RAB39B	c.503C > A missense mutation	[92]
RAB39B	c.557G > A missense mutation	[94]
RAB39B	c.574G > A missense mutation	[95]
RAB39B	c.432delA single base pair deletion	[96]
RAB39B	c. 536dupA duplication	[97]
RAB39B	c.543A > G; c.215 + 61G > ; c.215 + 39C > G	[98]
RAB39B	c.137dupT; c.371delA	[99]
	**Activity or pathological changes**	
Rab3a	Loss of coupling with rabphilin	[100]
Rab35	Increased levels in PD patient serum	[101]
Rab5	Accumulation in GCI	[102,103]
Rab1	Increased levels protect against aSyn-mediated neuron loss	[104]
Rab3a	Protects against aSyn-mediated neuron loss	[105]
Rab8a	Protects against aSyn-mediated neuron loss	[105]
Rab3a	Aberrant interaction with A30P aSyn	[107]
Rab5	Aberrant interaction with A30P aSyn	[107]
Rab8	Aberrant interaction with A30P aSyn	[107]
Rab8b	Promotes aSyn aggregate clearance	[108]
Rab11a	Promotes aSyn aggregate clearance/aSyn secretion	[108]
Rab13	Promotes aSyn aggregate clearance/aSyn secretion	[108]
Rab3a	Regulates aSyn binding to presynaptic membranes	[109]
Rab5a	Mediates aSyn endocytosis (spreading?)	[110]
Rab27b	Reduces aSyn spreading via nonexosomal pathways	[111]

**Table 2:** Alterations in Rabs in LRRK2-associated parkinsonism and the effect of Rabs on LRRK2 trafficking.

**Rab Alterations in LRRK2-Associated Parkinsonism**		
**Rab**	**Alteration Type**	**References**
	**Phosphorylation/activity changes**	
Rab8a	Aberrant phosphorylation by G2019S-LRRK2	[119]
Rab7L1	Aberrant phosphorylation by R1441C, Y1699C and G2019S-LRRK2	[120]
Rab10	Phosphorylation increase (R1441C)/decrease (G2019S)	[123]
Rab7	Decrease in protein activity induced by expression of PD-associated LRRK2 mutants	[124]
Rab8a	Impaired function mediated by G2019S-LRRK2	[126]
Rab35	Increased phosphorylation mediated by G2019S-LRRK2	[128]
**Rab impact on physiological and pathological** **LRRK2 trafficking**		
	**Trafficking changes**	
Rab29	Abnormal recruitment of R1441G/C and Y1699C-LRRK2 to the Golgi without affecting LRRK2 phosphorylation activity	[121,122]
Rab32	Regulates LRRK2 late endosomal transport	[130]

**Table 3:** Involvement of Rabs in autophagy and autophagy-related synaptic alterations.

Rabs in Autophagy and Autophagy-Related Synaptic Alterations		
Rab	ROLE	Reference
Rab39b	Autophagy activation and fusion of autophagosomes with lysosomes	[30]
Rab26	Directs SVs into pre-autophagosomal structures	[90]
Rab1	Autophagosome formation	[163]
Rab5	Autophagosome formation	[163]
Rab7	Autophagosome formation	[163]
Rab7	Fusion of autophagosomes with lysosomes	[163]
Rab8b	Autophagosome formation (non-canonical autophagy)	[163]
Rab8b	Autophagy-based unconventional secretory pathway	[163]
Rab9a	Autophagosome formation	[163]
Rab11	Amphisome formation	[163]
Rab23	Autophagosome formation	[163]
Rab24	Fusion of autophagosomes with lysosomes	[163]
Rab32	Autophagosome formation	[163]
Rab33b	Autophagosome formation	[163]
Rab7	Involved in autophagosome formation during mitophagy	[170]
Rab8a	Downstream target of PINK1	[171]
Rab8b	Downstream target of PINK1	[166]
Rab13	Downstream target of PINK1	[171]
Rab5	Directs SVs to autophagy	[181]
Rab35	Directs SVs to autophagy	[181]
Rab4	Autophagosome formation and maturation	[181]
Rab5	Autophagosome formation and maturation	[181]
Rab10	Autophagosome formation and maturation	[181]
Rab11	Autophagosome formation and maturation	[181]

## Data Availability

Not applicable.
